# Strain-level dissection of the contribution of the gut microbiome to human metabolic disease

**DOI:** 10.1186/s13073-016-0304-1

**Published:** 2016-04-20

**Authors:** Chenhong Zhang, Liping Zhao

**Affiliations:** State Key Laboratory of Microbial Metabolism and Ministry of Education Key Laboratory of Systems Biomedicine, School of Life Sciences and Biotechnology, Shanghai Jiao Tong University, Shanghai, 200240 China; SJTU-Perfect China Joint Center on Microbiota and Health, Shanghai, 200233 China

## Abstract

The gut microbiota has been linked with metabolic diseases in humans, but demonstration of causality remains a challenge. The gut microbiota, as a complex microbial ecosystem, consists of hundreds of individual bacterial species, each of which contains many strains with high genetic diversity. Recent advances in genomic and metabolomic technologies are facilitating strain-level dissection of the contribution of the gut microbiome to metabolic diseases. Interventional studies and correlation analysis between variations in the microbiome and metabolome, captured by longitudinal sampling, can lead to the identification of specific bacterial strains that may contribute to human metabolic diseases via the production of bioactive metabolites. For example, high-quality draft genomes of prevalent gut bacterial strains can be assembled directly from metagenomic datasets using a canopy-based algorithm. Specific metabolites associated with a disease phenotype can be identified by nuclear magnetic resonance-based metabolomics of urine and other samples. Such multi-omics approaches can be employed to identify specific gut bacterial genomes that are not only correlated with detected metabolites but also encode the genes required for producing the precursors of those metabolites in the gut. Here, we argue that if a causative role can be demonstrated in follow-up mechanistic studies—for example, using gnotobiotic models—such functional strains have the potential to become biomarkers for diagnostics and targets for therapeutics.

## Gut microbiome—a new paradigm for understanding metabolic diseases

Obesity and related metabolic diseases such as diabetes and cardiovascular disease represent a major public health threat to both developed countries, such as the United States, and rapidly developing countries, such as China and India [1–3]. China, for example, has more than one hundred million diabetic patients and nearly five hundred million people with pre-diabetes [4]. Metabolic diseases alone could overwhelm the public health and medical systems in these countries unless something substantial happens in the prevention and treatment of these diseases in the next decade.

Human beings are superorganisms consisting of not only our own cells but also up to ten times more microbial cells, most of which are bacteria residing in the gut. The gut microbiota consists of hundreds of individual bacterial species, each of which contains many functionally different strains with significant genetic diversity. Studies of the contribution of the gut microbiome to the onset and progression of metabolic diseases, particularly adiposity and insulin resistance, the two hallmark characteristics of various metabolic diseases in their early stages, have resulted in a paradigm shift in understanding the root cause of human metabolic diseases in the last decade or so, and may bring new hope to countries devastated by such diseases [5]. However, most of the evidence so far is associative in nature. Mechanistic studies, which are needed for demonstration of causality, are mostly attempted at a community level or taxon level higher than species, such as genus, family or even phylum [5]. Bacterial species or other higher taxa are arbitrarily defined taxonomic units for clustering and categorizing strains, each of which consists of genetically identical cell populations. Since bacterial strains, equivalent to individual plants and animals, are the genetically defined, basic functional units of the gut ecosystem, dissecting the contribution of the gut microbiome to human metabolic diseases must be carried out at the strain level. Identifying and understanding all relevant strains in the gut microbiota that may have mechanistically contributed positively (detrimentally) or negatively (beneficially) to the onset and progression of metabolic diseases can lead to the discovery of new biomarkers of predictive and diagnostic value, as well as new targets for effective interventions in humans.

We argue that, unless we can identify specific functional strains of the gut microbiome and understand mechanistically how each individually or in combination contributes to the onset and progression of metabolic diseases, the translation of new microbiome findings to clinical practice for diagnosis and therapeutics will be rather limited. We discuss how high-quality draft genomes can be assembled directly from metagenomic datasets to provide strain-level genetic data that can be correlated with disease-relevant variations of metabolites in samples such as urine, as an example of systems-level discovery approaches for identifying specific functional bacterial strains that may play a causative role in human metabolic diseases. These strains can then be isolated into pure culture and confirmed mechanistically as having a causative role in metabolic diseases using gnotobiotic animal models. This approach may help to move the microbiome field from association at the community or high-taxon level towards causality at the strain level. Such genomic- and molecular-level studies can eventually lead to the discovery of biomarkers and drug targets in the gut microbiome for clinical applications.

## Role of the gut microbiota in metabolic diseases

Excessive visceral fat deposition is a primary pathological condition underlying many forms of metabolic diseases. A seminal paper in 2004 reported that the gut microbiota might act as an environmental factor for regulating fat storage in the host [6]. Subsequently, the results of several studies pointed to the involvement of the gut microbiota in fat accumulation [5]. Germ-free mice are resistant to high-fat-diet-induced obesity [7]. Lean germ-free mice accumulated 60 % more fat after being colonized with a normal gut microbiota despite a reduction in their food intake after the conventionalization. Transplantation of gut microbiota from obese mice or humans induced significantly higher fat accumulation in recipient mice than transplantation of gut microbiota from lean donors [8, 9]. Removal of gut microbiota by using cocktails of broad-spectrum antibiotics prevented fat accumulation even in genetically obese mice, such as *ob/ob* mice or Toll-like receptor 5 knockout mice [10, 11]. It was found that gut microbiota may promote fat accumulation by reducing the expression level of genes required for fatty acid oxidation, such as *Fiaf* (encoding fasting-induced adipose factor) in the gut, and by increasing the activity of genes needed for synthesizing new fat, such as *Acc1* (encoding acetyl-CoA carboxylase 1) and *Fas* (encoding fatty acid synthase) in the liver [6]. In 2015, a study showed that depletion of the gut microbiota by antibiotics or in germ-free mice increased browning of white adipose tissue and reduced obesity in the mice, possibly via eosinophil infiltration, enhanced type 2 cytokine signaling and M2 macrophage polarization [12]. Thus, dysregulation of genes involved in host lipid metabolism may be an important mechanism by which the gut microbiome promotes excessive fat accumulation in obesity.

Insulin resistance, the other hallmark feature of metabolic diseases [13, 14], has been mechanistically linked to a low-grade, systemic, chronic inflammatory condition in mice and humans [15]. The gut microbiota has also been associated with insulin resistance in mice and humans. Germ-free mice are insulin sensitive but can become insulin resistant after being conventionalized with gut microbiota, particularly from obese mice [7]. In obese human volunteers, systemic insulin sensitivity was improved within 6 weeks after receiving gut microbiota transplantation from healthy donors [16]. Thus, an obesity-associated gut microbiota may work as a virulence factor in driving insulin resistance.

Endotoxin, a proinflammatory form of lipopolysaccharide (LPS), was shown to be able to induce inflammation followed by both adiposity and insulin resistance when subcutaneously injected into mice fed on a low-calorie diet for several weeks [17]. This was the first evidence that LPS, a microbial product from the gut microbiota, may be driving inflammation and contributing to fat accumulation and insulin resistance. These results indicated that some endotoxin producers in the gut microbiota may contribute to the proinflammatory condition and progression of insulin resistance in the host. Recent studies suggest a possible role for LPS in fatty liver disease [18] and obstructive sleep apnea [19]—an indication that inflammation sustained by microbial products such as LPS may drive more forms of metabolic disorders. Thus, compelling evidence from mouse and human studies supports a pivotal role of the gut microbiota in the onset and progression of metabolic diseases. However, it has been a great challenge for the field to identify all relevant members of the gut microbiota that are associated with the development of metabolic diseases, and to demonstrate their causative contribution to pathophysiological changes critical for disease initiation and progression.

When dissecting and demonstrating the causative contribution of relevant members of the gut microbiome to human metabolic diseases, we should follow the logic of Koch’s postulates, which were established for identifying a causative agent in an infectious disease, but adapt them to the polymicrobial nature of the role of the gut microbiome in human chronic diseases. Firstly, we should do microbiome-wide association studies, in which all members of the gut microbiome that are positively or negatively correlated with disease phenotype(s) need to be identified. Secondly, the associated members should be isolated into individual pure cultures or strains. Individual strains or their combinations should be inoculated into germ-free animals to reproduce at least part of the disease phenotype(s). Thirdly, the molecular mechanisms underlying causation should be established, from colonization of the gut to development of the disease endpoints. After fulfilling these rigorous protocols, these strains would be accepted as causatively contributing to human metabolic diseases. They then have the potential to be new biomarkers and drug targets for clinical applications [5].

High-quality association studies are critical for the successful identification of potential key players of the gut microbiome in metabolic diseases, which can then be followed by rigorous molecular-level mechanistic studies as the ultimate evidence for causality. We argue that association studies at the strain level are pivotal for reducing spurious correlations and identifying “real targets” for mechanistic studies.

## Bacterial species and strains in metabolic disease

### Bacterial functions are strain-specific

The gut microbial ecosystem consists of bacterial populations as individual members, each of which has genetically identical cells derived from the same parent cell [20]. Any two populations can be distinguished by at least one single nucleotide polymorphism, and they may have different adaptive functions in the ecosystem—for example, a point mutation in a drug resistance gene can make a mutant population survive a new round of antibiotic medication, while the wild-type may have been wiped out [21]. Bacterial populations, which have been isolated in pure culture or detected by partial or complete sequencing of their genomes, are defined as strains [22]. One strain is thus (at least partially) a known population in the gut ecosystem. In bacterial taxonomy, a “species” would contain individual strains, with up to 30 % difference in their genomic homology; that is, two strains in the same named bacterial species can be genetically more different than humans and mice, which have only about 10 % genomic difference [23]. Genomic sequencing of many strains in the same named bacterial species has already revealed this huge genetic microdiversity. In all 17 sequenced strains of *Escherichia coli*, 2200 genes were conserved. However, pan-genome prediction indicates that *E. coli* species may contain a reservoir of more than 13,000 genes [24]. Complete sequencing of 34 strains of *Lactobacillus paracasei* identified about 1800 orthologous genes (OGs) in its core genome, but 4300–4500 OGs in its pan-genome [25]. Ecological functions in the gut microbiome would thus be population-dependent. Any attempts to dissect the contribution of the gut microbiome to human metabolic diseases starting with microbiome-wide association studies must recognize that the disease-relevant functions of the gut microbiota may well be strain-specific.

### Potential bias in taxon-based analysis

Different structural patterns of the gut microbiota have been associated with metabolic diseases, such as the ratio between Firmicutes/Bacteroidetes, high gene count versus low gene count, or profiles of specific operational taxonomic units (OTUs) that are associated with progression of a particular disease phenotype [26–32]. Patterns of the gut microbiota associated with obesity and metabolic disorders have been sought at the individual OTU level (roughly at species level) up to phylum level in16S rRNA gene-sequencing-based analysis. However, species in the same taxon from genus up to phylum can show widely diverse relationships with a particular disease phenotype—some may be positively associated, some negatively, and others may not be associated at all [33, 34]. If a function is encoded in the “core genome” of a taxon, all members of that taxon should have that function. If the function is encoded in the pan-genome only, one or a limited number of members would have that function [35, 36]. It is thus a serious concern if we consider all species (OTUs) in a taxon as one group and seek associations at each taxonomic level, before we can be sure that all OTUs in the same taxon encode the same functions. However, we know that even within the same species, there is often high micro-diversity.

Recent developments in metagenomics have started to provide researchers with tools that can dissect the gut microbiome at the strain level [37–40]. For example, a recently developed canopy-based algorithm can be used to assemble high-quality draft genomes of predominant gut bacteria, based on the principle that if two genes are encoded in the same DNA molecule, their abundances across all the samples in which they can both be detected would be highly correlated to each other [41]. Individual non-redundant genes obtained from metagenomic datasets of many fecal samples can be binned into co-abundance gene groups (CAGs) if their abundances are highly correlated with each other. Genes in each CAG are potentially originally encoded by the same DNA molecule. Assembly of high-quality reads mapped to all the genes in the same CAG can generate high-quality draft genomes. This algorithm allowed researchers to get direct access to the genome variations of predominant bacteria in the gut microbiome. Because each genome represents one single population, this means that strain-level, genome-centric analysis is possible with metagenomic datasets. However, as mentioned earlier, any such genome/strain-level studies need to be confirmed by downstream mechanistic studies, ideally with the strain containing the genome in pure culture, to establish a gnotobiotic model of metabolic disease.

### Functional species and strains of the gut microbiota in metabolic diseases

In recent years, a number of functional species and strains have been identified in human metabolic diseases. Some of these may induce or aggravate the disease, while others may be protective.

We found one example of an obesity-inducing strain in a human gut opportunistic species, *Enterobacter cloacae*, which is known to cause bacteremia when translocated into the bloodstream of immune-compromised individuals [42]. In a volunteer with 174.9 kg initial bodyweight, this species was found to comprise nearly 30 % of the total gut bacterial populations. After taking a dietary intervention aimed at modulating the gut microbiota, this species was almost non-detectable in the gut and the volunteer lost more than 50 kg of baseline bodyweight over 23 weeks, along with recovery of all parameters of metabolic syndrome. A strain named B29 was isolated from the volunteer’s baseline fecal sample and was confirmed to be a member of the overgrowing species of *E. cloacae.* When inoculated into the gut of germ-free C57/B6 mice fed on a high-fat diet, B29 induced fully developed obesity phenotypes, including inflammation, adiposity and insulin resistance. B29 colonization was also shown to be able to reduce the expression level of *Fiaf* in the ileum and promote the expression of *Acc1* and *Fas* in the liver. B29-colonized mice fed on normal chow or germ-free control mice fed on a high-fat diet did not become obese. Only the combination of a high-fat diet and mono-association of B29 led to elevated endotoxin levels in the serum and systemic inflammation, and local inflammation in the liver and fat pads. This is the first reported example in which a single strain can induce fully developed obesity phenotypes in gnotobiotic mice. This strain was thus identified as an obesity-inducing “pathogen” by following the logic of Koch’s postulates.

Although a member of a bacterial species that can cause infectious diseases [43], *E. cloacae* B29 did not induce any notable septic symptoms even when directly injected into the bloodstream of specific-pathogen-free mice [42]. Genomic sequencing of B29 did not lead to the discovery of known virulence genes apart from genes involved in the LPS biosynthetic pathway. B29 is thus a non-infectious strain of this pathogenic species. B29 reached a stunningly high population level in the gut of its morbidly obese human host—more than 30 % of the total gut bacterial populations. This indicates that this strain has the genetic capacity to outcompete other members of the gut microbiota and become the predominant population. Reaching such a high population level would differentiate it from other LPS endotoxin producers in the gut in that it could make a substantial contribution to inflammation and obesity phenotypes.

It is still not clear why this population can reach such a high level without evoking an acute host immune system response. The patient was reported to have had a serious infection at 4 months old and had received heavy antibiotic medication, and started to gain weight after that incidence. One possibility might be that this strain had colonized the host’s gut so early in life that the host’s immune system developed tolerance to its colonization in the gut. Thus, at least three genetically encoded functions might be needed for a gut bacterium to be a causal agent in obesity development: (1) a virulence factor that can induce inflammation—in this case, the best candidate is LPS endotoxin; (2) the capacity to grow to a high population level in the complex gut ecosystem; and (3) the capacity to evade host immune surveillance so that a high population level can not only be reached but also be maintained in the gut ecosystem. However, all these need to be mechanistically tested. The gnotobiotic model, in which B29 alone or in combination with other members of the gut microbiota can colonize the intestine, represents an ideal system for future elucidation of the molecular mechanism of causation, from colonization by particular members of the gut microbiome to the development of a non-communicable disease such as obesity.

Hopefully, the identification of B29 as a potential pathogenic strain for obesity-related disease from the *E. cloacae* species, which usually induces infectious diseases, will serve as a good example to encourage researchers in the microbiome field to focus on strain-level diversity when their primary interest is to understand not only the association but also the causative functions of gut bacteria in human chronic diseases [5, 42].

Potentially beneficial strains in obesity have also been identified, isolated and validated in animal models. A strain of *Akkermansia muciniphila* has been shown to have a protective effect against obesity in both humans and mice [44, 45]. *A. muciniphila* was found to be negatively associated with obesity and type 2 diabetes in rodents and humans. Administration of viable cells of the strain *A. muciniphila* Muc^T^ (ATCCBAA-835) protected high-fat-diet-fed mice from developing metabolic syndrome, possibly via increasing intestinal levels of endocannabinoids that control inflammation, gut barrier integrity and secretion of gut peptides, including the antimicrobial peptide RegIIIγ.

In an association study involving 416 twin pairs, the Christensenellaceae family showed increased abundance in individuals with low body mass index (BMI). After being transplanted to germ-free mice, *Christensenella minuta* (DSM22607), a strain of the only cultured member of the family Christensenellaceae, reduced weight gain and altered the microbiome of recipient mice. The strain has been reported to produce short-chain fatty acids, but it is not clear whether this function contributes to its protective effect [46]. It is also not clear whether all the members of this family would have this protective function. For that, the genes encoding this beneficial function would need to be present in the core genome of all members of this family [47].

The discovery of *E. cloacae* B29 as a potential pathogenic strain for human obesity is not accidental. It built on prior evidence accumulated over many years in the field on LPS, inflammation and obesity in both animal studies and human epidemiological studies [5]. However, such a path to discovery is of limited efficiency. The human microbiome field requires many new forms of technologies for the systematic discovery of most, if not all, the potential key players of the microbiome that might contribute to human chronic diseases.

Gut bacteria contribute to human metabolic phenotypes by producing and delivering bioactive metabolites into the host systemic circulation [48]. Metagenomics can identify specific strains or populations that may have the genetic potential to produce such bioactive substances and to be involved in a disease phenotype. Whether a particular strain actually contributes to the disease needs to be confirmed with functional studies; that is, whether the bioactive metabolites were actually produced by these bacteria and transported into their hosts, and whether these metabolites were indeed responsible for the disease phenotype. Thus, one important strategy is to link a strain or genome with a particular metabolite involved in a disease process. An integrated metagenomics–metabolomics approach may well serve such needs for the field.

## Approaches for dissecting the functional contribution of the gut microbiome to metabolic disease

**Fig. 1:**
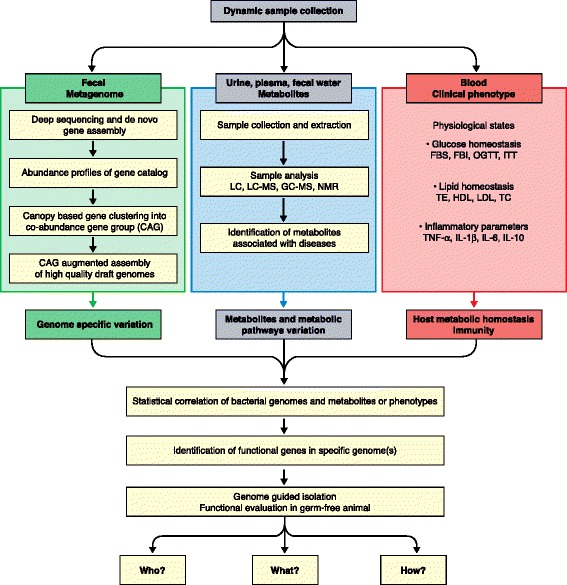
Integrated metagenomics–metabolomics approach for dissecting the strain-level contribution of the gut microbiome to human metabolic disease. Longitudinal, interventional experiments are accompanied by time-series and multisite sampling for capturing strain-level changes in the gut microbiota, and variations of host disease phenotypes and metabotypes. From blood samples, bioclinical parameters are obtained as measurements of changes in disease phenotypes. From the fecal samples, total DNA is extracted and shotgun sequenced. Genes assembled and identified in individual samples are then integrated to form a cross-sample, non-redundant gene catalog. The abundance profile of each gene in the catalog is assessed by counting the matching sequence reads in each sample. A canopy-based algorithm is used to cluster the large number of genes in the catalog into co-abundance gene groups (*CAGs*). Sequence reads from individual samples that map to the CAGs and their contigs are then extracted and used to assemble high-quality draft genomes, each of which is a strain or a group of highly similar strains. For the urine, plasma, or fecal water samples, metabolomic approaches such as nuclear magnetic resonance (*NMR*)-based metabolite profiling is used to capture variations in metabolites or host–bacteria co-metabolites. Variations in specific metabolites during the interventions or correlated with disease phenotypes are identified via multivariate statistics. Correlation analysis between these specific metabolites and prevalent genomes may lead to the identification of specific strains that harbor the genes needed to produce precursors of the disease-relevant metabolites or host–bacteria co-metabolites. These strains can be isolated based on their genomic information. Gnotobiotic animal models can be established by colonization with individual or combinations of these strains for mechanistic studies to validate and understand their causative roles in the development of metabolic disease phenotypes. Eventually, we may answer questions such as “Who?” does “What?” and “How?” regarding the role of the gut microbiome in human metabolic diseases. *FBI* fasting blood insulin, *FBS* fasting blood sugar, *GC–MS* gas chromatography–mass spectrometry, *HDL* high-density lipoprotein, *IL* interleukin, *ITT* insulin tolerance test, *LC* liquid chromatography, *LC–MS* liquid chromatography–mass spectrometry, *LDL* low-density lipoprotein, *OGTT* oral glucose tolerance test, *TC* total cholesterol, *TE* triglycerides, *TNF* tumor necrosis factor

Gut bacteria can produce various bioactive metabolites, which can enter the bloodstream of the host via the enterohepatic circulation or via a partially impaired gut barrier [48, 49]. One third of the small molecules in the bloodstream can be of gut bacterial origin [50]. Some of the bioactive metabolites can be detrimental to host health, such as those with cytotoxicity, genotoxicity or immunotoxicity [51–55]. When these toxic metabolites enter the bloodstream, they can contribute to the onset and progression of many forms of chronic diseases such as autism, cancer and diabetes [17, 56–59]. Notably, as a detoxification mechanism, these toxic metabolites can be further transformed by host liver enzymes into water-soluble derivatives that are excreted in the urine [57, 60]. Thus, one important strategy for identifying the species or strains of the gut microbiota that may be involved in the production of specific toxic metabolites could be to correlate species- or strain-level variations of gut bacteria with variations of metabolites in the urine and in other types of samples (Fig. 1).

### Integrating metagenomic and metabolomic approaches

In a proof-of-principle study, we collected urine and fecal samples from a four-generation, seven-member Chinese family over monthly intervals [61]. This time-series approach for the collection of both fecal and urine samples can help to capture intra-individual and inter-individual variations in both gut bacterial populations and urine metabolites to allow their correlation, to determine the functions of specific strains of the gut microbiota. Population changes of predominant bacteria were assessed by DNA fingerprinting and sequencing. Urine metabolites were profiled using ^1^H nuclear magnetic resonance (NMR) spectroscopy-based metabonomics. Although we could only identify a limited number of predominant bacteria with the fingerprinting technology, we achieved sub-species-level resolution of the predominant populations because this approach allowed two DNA fragments with a single nucleotide difference in their sequences to be resolved into two bands. A multivariate statistical method was used to correlate changes in the urine and fecal samples. This analysis led to the identification of ten bacterial populations, each of which showed a correlation with at least one urine metabolite. Two bacterial populations were identified as different strains of the species *Faecalibacterium prausnitzii*. One strain had associations with two urine metabolites, while the other strain had eight associations with urine metabolites—six positive associations and two negative ones. As a non-targeted discovery approach, this method opened new avenues for determining the functions of individual members of the microbiota [61].

Since the publication of this integrated metagenomics and metabolomics methodology, next-generation, high-throughput sequencing has revolutionized microbiome research. Metagenomic sequencing of total fecal DNA samples now enables researchers to access genomic information from gut bacteria that would otherwise be inaccessible using traditional culture-based technologies [62, 63]. At first, this genomic information can be used to profile variations at the individual gene level. Many studies have focused on functionally relevant genes that might be associated with host health or disease phenotypes [64–67]. Such a gene-centric approach for metagenomic data-mining has generated many new insights into the role of the gut microbiome in human metabolic diseases; for example, volunteers with a high gene count in their microbiomes seem to be better at responding to the same dietary intervention for controlling obesity than those with a low gene count [28, 68]. However, if millions of genes are identified from a metagenomic dataset, it is not technically feasible to correlate their changes with urine metabolome changes. Eventually, we still need to identify the genomic sequences of the strains in the gut microbiome that correlate with specific metabolites or disease phenotypes in order to understand the ecological interactions among them and between them and their hosts.

With this aim, we conducted a clinical trial of a gut microbiota-targeted dietary intervention during which urine and fecal samples were collected so that an integrated metagenomics–metabolomics strategy could be used to dissect the contribution of the gut microbiome to human metabolic disease [69]. Time-series sample collection in such a study design would increase the statistical power needed to correlate strain-level variations in the gut ecosystem with metabolites produced by gut bacteria and delivered into the host systemic circulation.

In this clinical trial, 17 morbidly obese children with a genetic defect called Prader–Willi syndrome were hospitalized for 3 months, and 21 children with simple obesity were hospitalized for 1 month, and both groups were placed on a diet based on whole grains, traditional Chinese medicinal foods and prebiotics. At baseline and at the end of each month, urine and fecal samples were collected. Both cohorts lost substantial amounts of their initial bodyweight and exhibited significantly improved glucose homeostasis, lipid profiles and liver function. Transplantation of the pre- and post-intervention gut microbiota from the same individual into germ-free mice showed that the pre-intervention microbiota induced inflammation in the gut and liver, and fat accumulation in adipocytes of the germ-free mice, whereas transplantation of the post-intervention microbiota did not induce these effects. 16S rRNA gene sequencing-based analysis also confirmed that the dietary intervention significantly modulated the gut microbiota structure of the volunteers, with concomitant improvement of metabolic phenotypes. To assess the contribution of the gut microbiome to childhood obesity in the two cohorts studied, we then used an integrated metagenomics–metabolomics approach to determine whether strain-level dissection could be achieved.

Metagenomic sequencing of 110 fecal DNA samples at 8 Gb each led to the identification of two million non-redundant genes. Using co-abundance analysis, 376 CAGs were obtained with more than 700 genes, indicating that they were bacterial genomes. Of these, 161 CAGs were selected for further analysis as they were shared by more than 20 % of the samples, and thus represented the predominant bacterial populations in these cohorts. From these 161 CAGs, 118 high-quality draft genomes were assembled, each of which could meet at least five of the six criteria for assessing the quality of Human Microbiome Project reference genomes obtained from sequencing of pure cultures.

After the dietary intervention, NMR-based metabolomic analysis of urine samples showed that the levels of four metabolites were significantly increased and the levels of nine metabolites were decreased. Interestingly, among the nine metabolites with decreased levels was trimethylamine-*N*-oxide (TMAO), a co-metabolite between host and gut bacteria, which can promote plaque formation and increase the risk for atherosclerosis. TMAO is transformed in the liver from a precursor called trimethylamine (TMA), which in turn is produced by some gut bacteria by fermenting dietary choline from animal fat such as phosphatidylcholine [70]. To determine which gut bacteria can convert choline into TMA, we used Spearman correlation to test the association between the 118 high-quality draft genomes and the urine concentration of TMAO. Among the 31 genomes that were correlated with TMAO concentration in the urine, 13 were found to contain the genes encoding choline TMA-lyase and choline TMA-lyase-activating enzyme, the two genes required to convert choline to TMA. These genomes are members of *Ruminococcus* spp., *Parabacteroides* spp. and *Bacteroides* spp. The next step would be to isolate these bacteria and validate their functions for converting choline to TMA and their association with increased risk of atherosclerosis in gnotobiotic models.

### The need for new integrative approaches

Since the publication of proof-of-principle studies to show the feasibility of using integrated metagenomics–metabolomics approaches for “functional metagenomics”, researchers have called for “a marriage between metagenomics and metabolomics”, not only in the human microbiome field but also in almost all other microbiome fields [71–76]. Such approaches are facilitating the identification of bacterial populations that are associated with functional effects in health and disease.

Integrated microbiome and metabolome analysis identified the genera *Ruminococcus* and *Butyricicoccus* as being associated with butyrate production, and distinguished elderly subjects in the community from those in long-term residential care [77]. Two-week food exchanges in subjects from two populations, in which African–Americans were fed a high-fiber, low-fat African-style diet and rural Africans were fed a high-fat, low-fiber Western-style diet, resulted in changes at the specific genus level of the microbiota and associated changes in metabolites in urine and fecal matter known to affect cancer risk [78].

Chromatographic–mass spectrometric methods, such as ultra-performance liquid chromatography–mass spectrometry (UPLC–MS)-, LC–MS-, and gas chromatography–mass spectrometry (GC–MS)-based profiling techniques, have also been widely used to detect metabolites in urine, plasma, or other samples [79, 80].

New approaches for the integration of microbiome and metabolomic profiles are also being developed. For example, Noecker and colleagues introduced a comprehensive analytical framework to systematically link variations in metabolomic data with microbial community composition [81]. Bouslimani and colleagues described the implementation of an approach to study the chemical make-up of the surface of human skin and to correlate this with specific skin microbes, using three-dimensional mapping of MS data and microbial 16S rRNA gene sequences [82]. However, strain-level dissection is still a bottleneck for many association studies based on these various approaches. The integrated metagenomics–metabolomics strategy described earlier can identify high-quality draft genomes, which are not only associated with disease-relevant metabolites, but are also shown to encode the genes required for producing the precursors of those metabolites. These identified genomes represent good candidates for downstream isolation and mechanistic studies in gnotobiotic models. Yet this approach has its limitations. For example, the canopy-based algorithm can only reconstruct high-quality draft genomes of prevalent gut bacteria. Furthermore, the NMR-based metabolomics method is also rather limited in identifying disease-relevant urine metabolites. Therefore, more universally applicable approaches are needed to link specific strains or populations in the microbiome with specific metabolites to facilitate strain-level dissection of the contribution of the gut microbiome to human metabolic diseases.

## Conclusions and future directions

Strain-level dissection of metagenomic datasets is crucial for conducting high-quality association studies as the first step for demonstrating a causative role for the gut microbiome in human metabolic diseases. However, many confounding factors may impair the quality of associative findings.

The genetic capacity of a functional microbial gene or pathway to contribute to a disease phenotype in the host does not necessarily lead to a causative interaction in the gut ecosystem. For example, the genomes of many bacterial strains in soil environments encode the pathway for converting choline to TMA [83]. We can envision that colonization of germ-free animals with such strains may lead to the associated disease phenotype, but such results may be spurious because these strains are not normal members of the gut ecosystem. Only TMA-producing strains resident in the human gut may have the potential to contribute to atherosclerosis.

Our Prader–Willi syndrome study [69] showed that among the 31 bacterial genomes that were positively associated with urine TMAO concentration, only 13 encoded the functional genes required to convert choline to the precursor TMA. This means that more than half of the associations may not be relevant for this function. Isolating the strains corresponding to the 13 genomes, that were not only correlated with urine TMAO concentration but also harbored the functional genes, would be the next logical step to move to mechanistic studies to investigate a causative role for these strains in the development of the disease phenotype.

Thus, direct assembly of high-quality draft genomes from metagenomic datasets, covering samples with sufficient inter-individual and intra-individual variations in bacterial populations, may transform human microbiome studies from mainly cataloging and inventory, to functionally demonstrating causative links between specific species or strains of the gut microbiota and defined pathophysiological processes in the host. Correlating fluctuations of these bacterial genomes in the gut with disease-relevant metabolites in samples such as urine, serum or fecal water can facilitate not only the identification of potentially important bacteria, but also the formulation of hypotheses on how they may impact host metabolism and participate in the pathology of chronic diseases. Findings from such studies have the potential to identify key functional bacterial strains in the gut microbiota as new diagnostic biomarkers and interventional targets for metabolic diseases.

## Abbreviations

BMI: body mass index
CAG: co-abundance gene group
GC–MS: gas chromatography–mass spectrometry
LC–MS: liquid chromatography–mass spectrometry
LPS: lipopolysaccharide
NMR: nuclear magnetic resonance
OG: orthologous gene
OTU: operational taxonomic unit
TMA: trimethylamine
TMAO: trimethylamine-*N*-oxide
UPLC–MS: ultra-performance liquid chromatography–mass spectrometry
